# Incidence of Parkinson’s disease in Germany based on prevalence data from 70 million patients of the statutory health insurance

**DOI:** 10.1186/s12883-024-03739-4

**Published:** 2024-06-27

**Authors:** Claudius Wattenbach, Gisa Ellrichmann, Insa Feinkohl, Sabrina Voß, Ralph Brinks

**Affiliations:** 1 Faculty of Health/School of Medicine, Chair of Medical Biometry and Epidemiology, Witten/Herdecke University, Witten, 58448 Germany; 2 Faculty of Health/School of Medicine, Chair of Neurology II, Witten/Herdecke University, Witten, 58448 Germany; 3 Department of Neurology, Klinikum Dortmund gGmbH, Dortmund, 44137 Germany; 4 Max-Delbrück Center for Molecular Medicine in the Helmholtz Association (MDC), Berlin, 13092 Germany

**Keywords:** Epidemiology, Parkinson’s disease, Illness-death model, Adolescents

## Abstract

**Background:**

Parkinson’s disease (PD) is a progressive neurodegenerative disorder characterized by motor and nonmotor system manifestations and psychiatric symptoms. The aim of this study was to estimate the age- and sex-specific incidence of PD in Germany using an illness-death model and a corresponding partial differential equation (PDE) based on prevalence and mortality data.

**Methods:**

Based on a PDE that describes the dynamics in an illness-death model, the age- and sex-specific incidence of PD in Germany was estimated using published prevalence and mortality rates. Prevalence rates were provided by the Central Institute for Statutory Health Insurance (Zi) for the period from 2010 to 2019. Parkinson’s related mortality was estimated based on comparable population data from Norway. Bootstrapping was used for incidence estimation (median of 5000 samples) and to obtain 95% confidence intervals to interpret the accuracy of the incidence estimation.

**Results:**

Men had higher incidences of PD than women at all ages. The highest incidences (median of 5000 bootstrap samples) for both groups were estimated for the age of 85 years with an incidence of 538.49 per 100,000 person-years (py) in men and 284.09 per 100,000 py in women, with an increasing width of bootstrapping 95% CIs showing greater uncertainty in the estimation at older ages.

**Conclusion:**

The illness-death model and the corresponding PDE, which describes changes in prevalence as a function of mortality and incidence, can be used to estimate the incidence of PD as a chronic disease. As overestimation of incidence is less likely with this method, we found incidence rates of Parkinson’s disease that are suitable for further analyses with a lower risk of bias.

## Introduction

Parkinson’s disease (PD) is the second most common neurodegenerative disease worldwide after Alzheimer’s disease.

The prevalence of PD increases with age. In women, it is 41 per 100,000 py in the 50–59 age group and 1,517 per 100,000 py in the 80+ age group. In men, it rises from 134 to 2,101 per 100,000 py in the same age groups [1]. In most cases, PD is diagnosed between the ages of 55 and 60 years and men are more frequently affected than women are. The lifetime risk of developing PD is 2% for men and 1.3% for women [2]. New preventive measures or treatment options are needed to prevent the aging of society from exacerbating this problem in the future [3].

Between 1990 and 2015, the number of people suffering from PD doubled, which may have been in part due to increased awareness and diagnoses as well as increased life expectancy and an aging population. The Global Burden of Disease study showed that PD was the fastest-growing neurological disorder worldwide in 2015 in terms of prevalence, physical impairment due to the disease and mortality [4]. Risk factors for the development of PD include a higher age; environmental factors; lifestyle (e.g. smoking, alcohol intake, coffee consumption, dairy product intake, farming, pesticides, rural living, and welding); drug/medical history (e.g. beta-blockers, calcium channel blockers, and ibuprofen); head injury; physical activity; and genetic predispositions (e.g. SNCA, LRRK2, VPS35, and Parkin) [3–6].

Projections of future prevalences and the expected numbers of patients with PD thus have a high degree of uncertainty. The illness-death model has already been used for the projection of future prevalence for other diseases, such as diabetes [7, 8]. These projections require a valid incidence estimation. Since the existing PD incidence estimation [9] is based on claims data needing disease-free intervals, which comes with a risk of an overestimation of incidence [10], we calculated the incidence of PD in Germany for the period from 2010 to 2019 based on a partial differential equation (PDE) [11] published by Brinks and Landwehr. Estimation of incidence based on PDE neither requires longitudinal cohort studies nor disease-free intervals so that overestimation of incidence is less likely with this method. The new Incidence estimation has a lower risk of bias. Here, we set out to estimate the incidence of PD in Germany from 2010 to 2019 based on the relationships between the incidence, mortality and prevalence of PD, as described in an illness-death model (IDM). In addition, this estimation of incidence will be compared with another method for estimating the incidence of PD in Germany [9].

## Methods

### Data

Our analysis uses prevalence data published by the Central Institute for Statutory Health Insurance (Zi) as “Care Atlas” (Versorgungsatlas) in 2022 [10]. This contains accounting documents from contract physicians for 2010 to 2019 for people with statutory health insurance who went to the physicians at least once in the respective year: In 2010, these included 69 million people; in 2019, the number increased to 71 million. Thus, the dataset covers more than 80% of the German population, and the dataset can be considered representative therefore we assume a low selection bias. Data was anonymized and contained, among other data, sociodemographic characteristics, comorbidities/diagnoses, billed services and medical history documented by the physicians. The population included people with a confirmed diagnosis of PD in at least two quarters within twelve months to exclude patients with only a suspected diagnosis of PD from the dataset. We use aggregated sex- and age-specific prevalence data published in [10]. These calculations used ICD diagnosis code ICD G20.-22 for the definition of PD: primary idiopathic Parkinson’s syndrome or secondary Parkinson’s syndromes (such as drug-induced PD or PD resulting from other underlying diseases). In line, our analysis is based on the same ICD codes. No information on the diagnosing person (i.e. general practitioner, neurologist etc.) were available.

Only anonymized data were used, so no approval by an ethics committee was needed [10–12]. The prevalence per 10,000 insured people was stratified by age, sex and region and the Zi data on the prevalence of PD in Germany for the years 2010 to 2019. Our analysis used prevalent cases for men and women (aged 20 to 109 years – as PD is less prevalent in individuals aged younger than 20 years) in Germany in 2010 and 2019. Of the 69 million people in 2010, 359,060 were diagnosed with PD (194,349 (54.1%) women and 164,711 (45.9%) men). Among 71 million people in 2019, 378,243 were suffering from PD diagnoses: 188,139 (49.7%) were female and 190,104 (50.3%) were male [10].

### Illness-death model

**Fig. 1:**
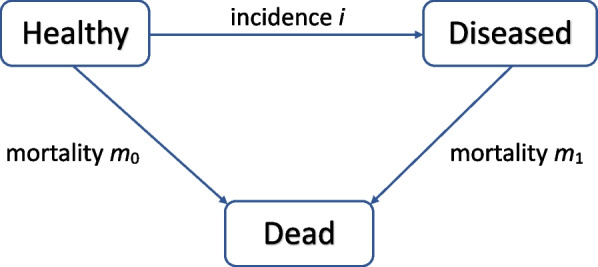
The illness-death model with mortality and incidence rates as transition intensities between states

Figure 1 shows the illness-death model (IDM) without recovery (Keiding) [13] that is a multistate model describing the dynamics and relationship of prevalence, incidence and mortality of chronic diseases using the three states “Healthy”, “Diseased” and “Dead”.

Transitions between the states are the incidence rate *i* (*Healthy* to *Diseased*) and the mortality rate of healthy *m*_*0*_ (*Healthy* to *Dead*) and diseased individuals *m*_*1*_ (*Diseased* to *Dead*). These transitions are irreversible, as we assume a chronic disease without recovery (no transition back to a previous state). The transition rates are dependent on two times: age in years (*a*) and calendar time (*t*) (*i*(*t,a*)*, m*_*0*_(*t,a*)*, m*_*1*_(*t,a*)). Brinks and Landwehr [12] introduced a partial differential equation (PDE) that describes the relation between the transition rates and the prevalence of the related chronic condition. With the following PDE changes in prevalence (p) can be described depending on the rates in the IDM (*i, m*_*0*_*, m*_*1*_):

$$\left(\frac{\partial }{\partial t}+\frac{\partial }{\partial a}\right)p=\left(1-p\right)\left[i-p\left({m}_{1}-{m}_{0}\right)\right]$$

Using the general mortality $m = p{m}_{1}+(1-p){m}_{0}$ and the mortality rate ratio $MRR =\frac{{m}_{1}}{{m}_{0}}$, this can be changed to:

1 $$i=\frac{\left(\frac{\partial }{\partial t}+\frac{\partial }{\partial a}\right)p}{1-p}+m\frac{p\left(MRR-1\right)}{1+p\left(MRR-1\right)}$$

This formula can be used to calculate the incidence rate of chronic disease under consideration.

### Statistical analysis

For the calculation of the incidence using the PDE in formula (1), prevalence data for PD (from 2010 and 2019) and the mortality in the population, as well as the mortality rate ratio (MRR) of diseased and healthy patients, i.e., the ratio of mortality rates of diseased over non-diseased patients, are needed. We extracted the prevalence data for PD from the Zi dataset (from 2010 and 2019). Data on general mortality in the German population are taken from “The Human Mortality Database” (HMD). As mortality rate ratios are stable across populations [14], the missing MRR for PD in Germany was replaced by the MRR from a suitable and comparable population from a Norwegian study [11] that was identified with a literature search in MEDLINE. The study used data from the Norwegian Central Registry to calculate the incidence, prevalence and mortality of PD in the Norwegian population for the period from 2004 to 2017. According to the law of total probability *m* = *p m*_1_ + (1-*p*) *m*_0_ can be solved by m_0_ resulting in *m*_0_ = (*m*—*p m*_1_)/(1-*p*). Thus, we were able to calculate the age- and sex-specific MRRs (MRR = m_1_/m_0_) stratified for men and women using the mortality for the population with PD and the general mortality. In our analysis, age was limited to the ages from 20 to 109 years and calendar time to the period from 2010 to 2019. Therefore, the prevalence data collected by Dammertz et al. [10] for the years 2010 and 2019 were used for the calculation of the incidence for the same period. Using the general mortality (*m*) in Germany and MRR from Norway, formula (1) was used to calculate the incidence rate. The estimation of incidence was performed via calculation of median age-specific incidences of bootstrap samples. Bootstrap samples (stratified by sex and age) are based on the underlying prevalence and mortality of our analysis. Based on ages from 20 to 90 years (with 5-year step size) and year 2014.5 (the mathematical mean between 2010 and 2019) we used uniformly distributed random numbers to draw random samples from these input data. For each of these samples, the corresponding incidences were estimated using the PDE (formula (1)). To test the uncertainty and accuracy of the estimated incidence, 95% bootstrap confidence intervals were calculated. To determine the age-dependent 95% bootstrap confidence intervals, the 2.5% and 97.5% quantiles were determined (stratified by sex). We used a total of 5000 different bootstrap samples in accordance with the recommendations for resampling in Efron and Tibshirani [15].

All calculations were performed using the freely available statistical software R version 4.1.3 (The R Foundation for Statistical Computing).

## Results

### Input data on PD prevalence

**Fig. 2:**
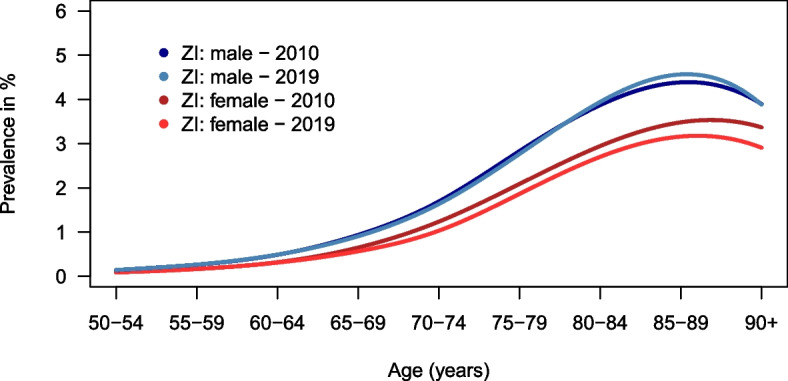
PD prevalence in Germany for 2010 and 2019 stratified by age and sex

Figure 2 shows the prevalence of PD calculated with Zi-data. Prevalence increased from 55 to 59 years of age and reached a maximum at 85 to 89 years of age for both men and women.

### Age- and sex-dependent incidence rate of PD

**Fig. 3:**
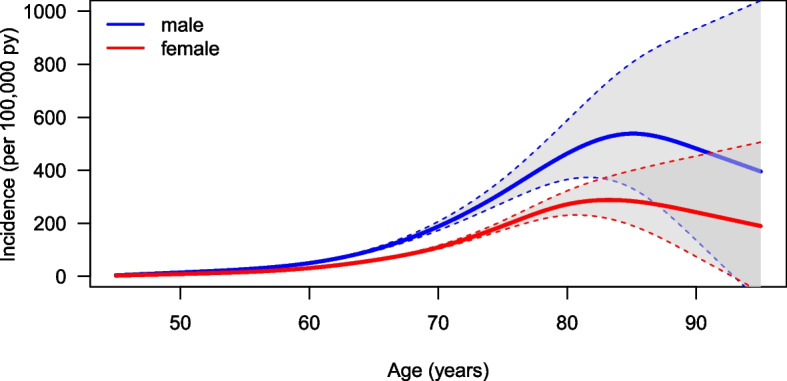
Median age-dependent incidence rate for 5000 bootstrap samples (solid line) and 95% bootstrap confidence intervals (dashed lines) for men (blue) and women (red)

Figure 3 shows the age-dependent incidence rate for PD stratified for men and women per 100,000 person-years (py). Men had higher incidences (median of 5000 bootstrap samples) for all ages. The incidence rate increased from 55 years of age and reached a maximum at the age of 85, with approximately 537,87 per 100,000 py for men and approximately 283,49 per 100,000 py for women. From the age of 85, the incidence decreased.

### Analysis of the accuracy of incidence estimation

To analyse the accuracy and variability in the incidence estimation, we calculated 95% bootstrap confidence intervals based on the 2.5% and 97.5% quantiles of the estimated incidence in 5000 random samples. Figure 3 shows these age-dependent intervals stratified by sex. In addition, the age-dependent median in the bootstrap samples is shown.

In the younger age groups, the variability in incidence was low. In addition, there were only minor differences between men and women. With increasing age, the median incidences increased. This was parallelled by an increased uncertainty of the estimation, as evidenced by wider confidence intervals starting at approximately 75 years of age. This is due to the input data and the indirect estimation method. Mortality in the older age groups becomes increasingly imprecise, as the Norwegian mortality rates show very large error bars here. For those aged 85 and above the intervals for men and women are overlapping. The median incidence in women is less than the median incidence in men for all age groups. The difference between men and women increases with age. As the incidence of PD for the age groups under 50 is very low and difficult to recognize graphically, we have decided not to include it in the graphs. These data can be found in the following Table 1:

**Table 1:** Calculated incidence of PD in the period from 2010–2019 for males and females with 2.5% and 97.5% quantile rounded to three decimal places

**Incidence of PD in Germany per 100,000 py**				
**Age (years)**	**Males**	**95%-CI**	**Females**	**95%-CI**
**20—24**	0.192	[0.182; 0.202]	0.188	[0.185; 0.191]
**25—29**	0.358	[0.350; 0.366]	0.370	[0.364; 0.375]
**30—34**	1.018	[1.012; 1.025]	0.688	[0.684; 0.691]
**35—39**	2.383	[2.342; 2.426]	1.372	[1.350; 1.393]
**40—44**	3.656	[3.589; 3.727]	2.587	[2.550; 2.625]
**45—49**	6.819	[6.729; 6.924]	4.552	[4.516; 4.591]
**50—54**	14.581	[14.242; 14.941]	8.831	[8.673; 8.996]
**55—59**	26.867	[26.055; 27.802]	16.142	[15.780; 16.525
**60—64**	50.080	[48.281; 52.267]	31.142	[30.514; 31.878]
**65—69**	100.070	[94.820; 106.463]	60.766	[58.793; 63.057]
**70—74**	188.074	[173.123; 206.205]	109.328	[103.411; 116.028]
**75—79**	316.664	[276.168; 365.991]	190.175	[174.146; 209.156]
**80—84**	463.150	[363.369; 586.325]	271.591	[229.452; 322.184]
**85—89**	537.868	[327.327; 796.870]	283.491	[189.851; 396.085]
**90 + **	480.360	[127.055; 922.867]	241.091	[69.568; 448.543]

## Discussion

### Summary

The incidence of PD for men and women in Germany between 2010 and 2019 was estimated based on prevalence and mortality data using the illness-death model for PD (as a chronic condition) and a corresponding partial differential equation. The incidence of PD in men was higher than that in women at all ages. Both had a maximum at the age of 85, with an incidence of 537.87 per 100,000 py for men and 283.49 per 100,000 py for women. Before the age of 85, the incidence increased quickly; after that age, a decrease was seen. The variability in the estimation was investigated using 95% bootstrap confidence intervals based on 5,000 bootstrap samples, with a strongly increasing uncertainty at older ages.

The possibility of using the new method with a PDE, that describes the illness-death model to estimate the incidence, was used for the estimation of the incidence for other chronic conditions like type 1 and type 2 diabetes [16]. These studies already showed advantages of this new method. The PDE-method that is new for estimating the incidence of PD offers the possibility of incidence-estimation based on prevalence data. This shows that incidence studies (large cohort studies) can be avoided by using the PDE. The new method presented in this work can both reduce costs and save time [16]. Additionally, an overestimation of incidence resulting from the use of disease-free intervals with another method [9], can be avoided.

### Comparison with the Zi estimation

**Fig. 4:**
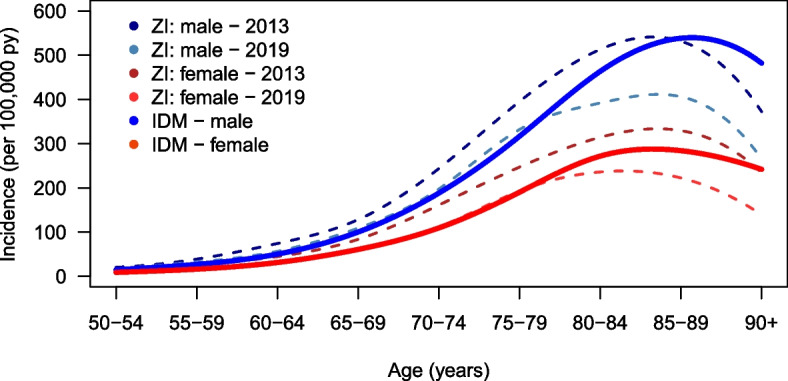
Comparison of the Zi incidence with that estimated by the PDE. The Zi incidence data were categorized for the age groups shown above. The incidence estimation using PDE was calculated for the age range of 50–90 years

In another publication from the authors of the Care Atlas for PD, they also assessed an incidence of PD in Germany for 2013 and 2019 [9]. The incidence in men peaked in 2013, with approximately 531 new cases per 100,000 insured persons in the 85–89 age group and approximately 406 new cases per 100,000 in 2019. There were approximately 329 new cases per 100,000 insured persons among the 85–89-year-olds in 2013 and approximately 235 in the 80–84-year-old age group in 2019. In most age groups, the incidence was slightly higher than the incidence in our analysis, in which the average incidence was estimated for the years 2010 to 2019. For men over 85 years of age, our incidence estimate is partly higher than that of the Zi. As mentioned above, this is most likely due to the high uncertainty of our estimation in these age groups. Figure 4 shows the visual comparison of the incidence estimated by Dammertz et al. [9] with our incidence estimate. Abbas et al. [17] showed that incidence estimations based on claims data such as the one presented in Dammertz et al. [9] need disease-free intervals with larger intervals, to enable an incidence estimate. The need for disease-free intervals leads to the potential for extreme overestimation of the incidence [17]. Consequently, the differences are based on the fact that an overestimation was presented in Dammertz et al. [9] resulting from claims data as the basis for analysis. In addition, the differences can also be explained by the fact that the incidence estimates are based on data from the outpatient and contract medical sectors; therefore, the exact time of diagnosis may be questionable. Due to irregular visits to medical doctors, diagnoses could be delayed. As the Zi collects prevalence data quarterly, incident patients are potentially recognized too late. This could bias the incidence recorded by the Zi [17]. In addition, diagnosis-free quarters lead to a Neyman bias (also known as prevalence-incidence bias). This bias occurs when the incidence of a disease is estimated from relatively short observation periods of prevalence. A consequence of this bias is an overestimated incidence caused by the situation where already prevalent cases are incorrectly categorized as new incidents [18, 19].

### Comparison to literature

The Global Burden of Disease Study [20] found an incidence rate of 381.5 per 100,000 py in Germany in 2015 with a 95% confidence interval of 350.48 to 418.85 for men and 222.47 with a 95% confidence interval of 189.64 to 254.98 for women. Compared to studies from other European countries [11, 21–24], the incidence of PD that we estimated in Germany was both higher [11, 21, 23, 24] and lower [22] in older age groups. Brakedal et al. [1] reported comparable incidences in the age group of 80–84 years in Norway, with 213 for men and 130 per 100,000 insured persons for women. For the diagnosis of PD, the authors used, among other factors, the prescription of PD specific drugs over a period of at least 180 days. This data is based on the “Norwegian Drug Prescription Database” (NorPD), a central register in Norway in which the dispensing of all prescription drugs, including the ICD diagnosis code, is recorded. In comparison to our data basis, this allowed a more precise definition of PD. Blin et al. [21] used the national healthcare insurance database (EGB) of France to describe the incidence of PD. At the time of the study, the database covered approximately 74% of the French population. They found incidence rates between 442 and 560 per 100,000 py for men aged 85 years and older in France. The incidence rates among women in the same age group ranged from 251 to 313 per 100,000 py. These large ranges are due to different definitions of a case. Okunoye et al. [22] also used different definitions of cases for the estimation of PD incidence in the UK. Depending on the definition, incidences of approximately 140 to 330 per 100,000 py were obtained in the age group from 80 to 89 years. Their data source was the IQVIA Medical Research Data (IMRD), which is based on data from more than 700 general practitioners and covers 6.2% of the UK population. The problem with the different case definitions in Blin et al. and Okunoye et al. is similar to our data source from Zi. The diagnosis of PD is too broad, which can falsify the results. PD is mixed with secondary Parkinson syndromes such as parkinsonism and parkinsonoid, that are not related to PD itself. Pupillo et al. [23] analysed the prevalence and incidence of PD from a nationwide database from general practitioners in Italy. The incidence rates per 100,000 py were 142.7 for men and 101.6 for women among the 75- to 84-year-old patients. One limitation of this study was that they had a passive reporting system that may suffer from underreporting of cases. Both in Okunoye et al. and in our data basis, there is a higher probability of misclassification of PD because the diagnosis was made by general practitioners rather than by neurology specialists. Sipilä and Kasinen [24] reported incidence rates of approximately 150 to 250 in men over 80 years of age and approximately 80 to 120 in women over 80 years of age in Finland. In this study, 70- to 79-year-old patients had higher incidence rates among women. Again, the prescription of PD specific medication was used to identify PD cases.

Outside Europe, Park et al. [25] described the incidence of PD in Korea in 2010 and 2015, which was approximately 210 (in 2010) and approximately 320 per 100,000 py (in 2015) in the group of 80-year-old men. The incidence in women was 180 (in 2010) and 280 (in 2015) per 100,000 py. Willis et al. [26] examined the incidence of PD in different populations in North America and reported that at 6,866,623 py in the population of people who were insured by Medicare, the incidence of PD was approximately 440 in men and approximately 240 per 100,000 py in women. In addition to different approaches to collecting or estimating the incidence of PD, there are also cultural factors in different countries that influence the likelihood of PD being diagnosed. For example, the severity of symptoms varies depending on the country and culture [27].

### Limitations

Our study has several limitations related to the databases used:

We used the 2.5% and 97.5% quantiles of the incidences calculated in 5000 samples as 95% bootstrap confidence intervals for the estimated age- and sex-specific incidence. Due to the uncertainty of the underlying mortality rate ratio for PD in the underlying Norwegian Central Registry, the estimation of incidences had increasing uncertainty, shown as rapidly widening confidence intervals (from approximately 80 years of age). This can be explained by the small number of patients in older age groups in the Norwegian database, as the confidence intervals are not based on the prevalence data of PD, with 70 million insured persons and more data in the older age groups.

A second limitation is that our data (Zi) contain complete information of all 70 million people with statutory health insurance in Germany (GKV only), but data from people with private health insurance (approximately 10 million people from PKV) are missing. Consequently, the data do not represent the whole German population (as was the case in the Norwegian Central Register), resulting in the possibility of selection bias. For the analysis, we assume that the proportion of people insured with private health insurance can be considered sufficiently small to neglect the potential for selection bias. We use aggregated sex- and age-specific prevalence data published in [14]. The ICD diagnosis code ICD G20-22 were used for the definition of PD (inclusion of ICD codes G21.x and G22* in addition to primary PD classified as G20.x.). As we only have these calculations and the aggregated data in Dammertz et al. [10], our analysis had to be based on the same ICD codes and it was not possible to estimate the incidence of PD for the ICD G20.x alone. As a result, PD prevalence is a combination of primary and secondary Parkinson syndromes such as parkinsonism or parkinsonoid and the inclusion criteria are too broad.

The use of these ICD codes can lead to a bias with the potential of higher incidence and prevalence estimations. Dammertz et al. [10] state that in 2010 90,2% and in 2019 91,4% of the diagnoses were G20: more than 90% of the prevalent cases used in our calculation are caused by diagnoses with ICD code G20.

The bias in our estimation caused by usage of the additional ICD codes (G21 and G22) is therefore present but can be assumed as minor. In addition, the data in [10] does not contain the diagnoses of people without statutory health insurance and people with undiagnosed PD that leads to the possibility of underestimating the prevalence and incidence.

The data set of the Zi was created using billing data from contract physicians of the statutory health insurance. However, the diagnosis codes were not only assigned by neurologists, but also by general practitioners. 95% of patients were treated by general practitioners during the observation period of the Zi, and more than 60% of them were also treated by neurologists – this might alter the diagnostic reliability.

The Zi dataset has potential further limitations, with implications for the analysis presented. The data are based on billing data from contract doctors of the Associations of Statutory Health Insurance Physicians only. Therefore, upcoding cannot be completely ruled out [28]. Since physicians are compensated for assigning different diagnostic codes, there is a possibility that a diagnosis in a patient’s record may be confirmed for a benefit in billing, even if the diagnosis of the corresponding disease is potentially not confirmed at that time and rejected afterwards. As the data collection was outpatient, this limitation is considered negligible. In an inpatient data collection, upcoding would be more possible due to the economic interests of large healthcare providers [29, 30].

### Conclusion

This study was the first to estimate the incidence of PD for men and women in all age groups ranging from 20 to 100 years in Germany between 2010 and 2019 based on data from 70 million insured persons and using the illness-death model and a related partial differential equation. The results showed that men had a higher incidence of PD than women, regardless of age. The highest incidence was observed in the 85–89 age group. The accuracy of the estimates was tested using 95% bootstrap confidence intervals, which showed increasing imprecision in the older age groups. Further research on mortality and PD incidence surveys is needed to obtain a more accurate picture of the incidence of PD and to better understand the associated challenges.

## Data Availability

The data are available in a public, open access repository. Data and source code for use with the open-source statistical software R (including data and analysis) for estimation of the incidence of PD in Germany are freely available on Zenodo (see reference and link below). Prevalence input is based on a large German claims dataset and is uploaded as aggregated data that are publicly available and can be downloaded free of charge from the Care Atlas (Versorgungsatlas) website. Data and source code (Zenodo): Wattenbach C, Voß S, Brinks R. Incidence of Parkinson’s disease in Germany based on prevalence data from 70 million patients of the statutory health insurance. 2024; 10.5281/zenodo.10795211; 10.5281/zenodo.10795211. Versorgungsatlas: https://www.versorgungsatlas.de/themen/alle-analysen-nach-datum-sortiert?tab=6&uid=121

## Abbreviations

PD: Parkinson’s disease
PDE: Partial differential equation
Zi: Central Institute for Statutory Health Insurance (Zentralinstitut für die kassenärztliche Versorgung in der Bundesrepublik Deutschland)
IDM: Illness-death-model
MRR: Mortality rate ratio
HMD: The Human Mortality Database
py: Person-years
GKV: Statutory health insurance (gesetzliche Krankenversicherung)
PKV: Private health insurance (private Krankenverischerung)
